# Perceived effectiveness of video interviews for orthopaedic surgery residency during COVID-19

**DOI:** 10.1186/s12909-022-03623-0

**Published:** 2022-07-22

**Authors:** Jonathan R. Warren, Lafi S. Khalil, Alexander D. Pietroski, Gabriel B. Burdick, Michael J. McIntosh, Stuart T. Guthrie, Stephanie J. Muh

**Affiliations:** Department of Orthopaedic Surgery, Henry Ford Hospital, 6777 W Maple Road, West Bloomfield Township, Detroit, MI 48322 USA

**Keywords:** Video interviews, Virtual interviews, Residency interviews, Orthopaedic surgery, Orthopedic surgery, Video, Virtual, Education, Perceptions, COVID-19

## Abstract

**Background:**

During the 2020–21 residency interview season, interviews were conducted through virtual platforms due to the COVID-19 pandemic. The purpose of this study is to assess the general perceptions of applicants, residents and attendings at a single, large, metropolitan orthopaedic residency with regards to the video interview process before and after the interview season.

**Methods:**

Surveys were sent to all orthopaedic applicants, residents, and attendings before the interview season. Applicants who received interviews and responded to the first survey (46) and faculty who responded to the first survey (28) were sent a second survey after interviews to assess how their perceptions of video interviews changed.

**Results:**

Initially, 50% of applicants (360/722) and 50% of faculty and residents (28/56) responded before interview season. After interviews, 55% of interviewees (25/46) and 64% of faculty and residents (18/28) responded. Before interviews, 91% of applicants stated they would prefer in-person interviews and 71% were worried that video interviews would prevent them from finding the best program fit. Before interviews, 100% of faculty and residents stated they would rather conduct in-person interviews and 86% felt that residencies would be less likely to find applicants who best fit the program. Comparing responses before and after interviews, 16% fewer applicants (*p* = 0.01) perceived that in-person interviews provide a better sense of a residency program and faculty and residents’ perceived ability to build rapport with interviewees improved in 11% of respondents (*p* = 0.01). However, in-person interviews were still heavily favored by interviewees (84%) and faculty and residents (88%) after the interview season.

**Conclusions:**

In-person interviews for Orthopaedic Surgery Residency are perceived as superior and are preferred among the overwhelming majority of applicants, residents, and interviewers. Nevertheless, perceptions toward video interviews improved in certain domains after interview season, identifying potential areas of improvement and alternative interview options for future applicants.

## Background

Medical education continues past the achievement of a medical degree in the form of residency. Residency is considered the most vital and important part of a doctors’ training, when medical knowledge is guided by mentors and expanded by attending faculty with a full career of experience to polish a compassionate, empathetic, evidence-based physician in training. Success in the rigorous residency training environment takes more than tangible knowledge. It requires trainees to dive deep in the intangible aspects of academic medicine, to corroborate with their peers, rely on their faculty, debate and challenge, investigate and research, and grow as a team of young physicians. The ability to thrive in this environment optimally relies on fitting in with the environment, the people, and the culture. Hence, the most common piece of advice given to medical students interviewing for residency positions is often to “find your fit” with the people who will train you [1, 2].

Historically, competitiveness for residency programs is gauged by the cognitive domains apparent in a student’s application as well as noncognitive skills that are best assessed during interviews [3–6]. Typically, noncognitive skills such as an applicant’s ability to communicate and develop interpersonal relationships, how mature and honest they are, and their overall interest in the field are easily palpable during interviews [3–6]. As such, the formal interactions that programs have with interviewees bear significant weight when program directors and other faculty select applicants for their program [7]. Likewise, informal interactions with applicants at recruitment events outside the interview give programs and students vital information in how compatible they are for each other [3, 5, 8–11]. Programs often evaluate whether applicants fit with the general culture, hold similar values and goals, interact well with faculty, and share similar interests with current residents. This compatibility or perceived fit of both parties for one another is often taken into great consideration during the selection process and overall plays a large role in how applicants and programs are ranked [12].

In light of the COVID-19 pandemic, in March of 2020 the Association of American Medical Colleges (AAMC) recommended that all residency interviews be conducted in a video setting for the 2020–2021 residency interview season [3]. Theoretically, there are a few notable advantages of video interviews compared to in-person interviews. Namely, they save time, reduce cost for applicants and programs, are overall more convenient, reduce applicant time spent away from school, and allow applicants to apply and interview at more programs [3, 6, 13–19]. Several studies have suggested that applicants and programs are receptive to video interviews as a viable alternative to in-person interviews [3, 13, 17, 18]. Conversely, applicants value in-person interviews as a way to gauge the morale of residents and gain insight to any program weaknesses, [20, 21] which are more apparent in-person [6]. Additionally, historical shortcomings from the perspective of programs include reduced ability to evaluate candidates for compatibility and personality, which are major factors that go into resident selection [3, 6].

The video platform of the 2020–2021 interview season has introduced elements to the residency interview process that will likely persist or be offered as an alternative moving forward. Gathering data on this new era of residency interviewing will provide indispensable information for the evolution of future residency interview seasons. The purpose of this study is to assess the general perceptions of applicants, residents and attendings at a single, large, metropolitan orthopaedic residency with regards to the video interview process before and after the interview season.

## Materials and methods

This was a survey study investigating the perceptions of medical school applicants, residents, and faculty regarding the new video platform universally utilized for residency program interviews during 2020–2021. Institutional review board (IRB) approval was granted by the main institution for this study (IRB# 14220). Additionally, the American Association of Medical Colleges (AAMC) granted approval for investigators to contact medical student applicants for participation in this survey study. Participants of this survey study were included if they applied to the orthopaedic surgery residency program at the main institution, or if they were a resident or attending physician within the orthopaedic surgery department at the main institution. Participants were excluded if they were not an applicant, resident, or attending of the orthopaedic surgery department at our academic hospital; they were unwilling or unable to comply with consenting for being surveyed; or they were unable to read or speak English. All methods for this study were carried out in accordance with the relevant guidelines and regulations of the IRB at the main institution and the AAMC. Additionally, informed consent was obtained from all subjects who participated in this study.

Four total Qualtrics surveys were created (Provo, UT). Two of these surveys were specifically designed for medical student applicants to complete before and after interviews. The remaining two surveys were designed for residents and attendings to complete, one before interviews and one after interviews. After receiving approval from the AAMC, email addresses belonging to all applicants to the orthopaedic surgery residency program at the main institution were extracted from the Electronic Residency Application System (ERAS). All 722 applicants were emailed individually and sent an invitation to participate in this study along with a brief study description. At the time of this first email, applicants were also sent a consent form to be read before completing the initial survey before interviews. All survey responses completed prior to the beginning of interviews in November 2020 were collected in a de-identified database. A similar process was conducted to deliver the surveys to residents and attendings prior to interview season, although emails were obtained through the secure email system at the main institution rather than ERAS.

Interviews at the main institution were conducted in December 2020, January 2021, and February 2021. Following interviews, in February–March 2021, all applicants who responded to the survey before interviews and who were interviewed by the main institution (46 of 360) were emailed with the after-interview survey. Similarly, all residents and attendings who responded to the initial survey (28 out of 56) were emailed their respective after-interview survey.

Surveys were composed of previously validated questions [22] as well as additional questions that aligned with goals of the study. Questions designed to assess participant perceptions utilized either a 5-point Likert scale (Strongly Disagree, Disagree, Neutral, Agree, Strongly Agree) or true/false format. All questions were verified and validated by the institutional review board, one senior orthopaedic surgery resident, and two orthopaedic surgery faculty with administrative positions within the department.

Questionnaire responses before and after interviews were compared to determine whether perceptions of the video interview platform changed among applicants, residents, and faculty. SPSS software was used for all statistical analyses (IBM Corp. Released 2017. IBM SPSS Statistics for Windows, Version 25.0. Armonk, NY: IBM Corp.). Significance was set at *p* < 0.05. Categorical variables were reported as frequency and percentages. Descriptive statistics were used to analyze continuous variables (median with range when not normally distributed and mean ± standard deviation when normally distributed). Normality was assessed using the Shapiro-Wilk test. Spearman’s rank correlation coefficient was used to describe the relationship between two continuous variables when normality was violated. Two-sample F-test for variance was used with a Student’s t-test to determine significance for normal data.

## Results

A total of 360 applicants and 28 residents and attendings responded to the surveys. Response rate to the applicant survey before interviews was 50% (360/722) and after interviews response rate was 55% (25/46). Among faculty, 50% (28/56) responded to the first survey and 64% (18/28) responded to the after-interview survey. Demographics of the responders are presented in Table 1.

**Table 1:** Demographics

	Male, n (%)	Age Range	Race
Applicants	360 (78%)	< 23 (0.55%)	White (63%)
		24–25 (21%)	Asian (20%)
		26–28 (57%)	Other (10%)
		29–31 (14%)	Black (6%)
		> 32 (7%)	Indian (1%)
Residents/Attendings	28 (56%)	25–30 (57%)	White (86%)
		31–35 (18%)	Asian (10%)
		36–40 (4%)	Other (4%)
		41–45 (11%)	
		61–70 (7%)	
		> 70 (4%)	

Prior to interviews, 91% (320/360) of applicants and 100% (28/28) of residents and attendings indicated that they prefer an in-person interview format compared to video interviews. Most applicants expressed initial concerns regarding the video interview format and were worried about their ability to represent themselves, find the best program, and successfully match (Table 2). Eighty two percent (23/28) of residents and attendings mirrored this worry about applicant ability to represent themselves in video interviews. A majority (95%, 320/360) of applicants indicated that in-person interviews would give a better representation of the culture of a residency program. Most residents and attendings agreed that video interviews would impact their ability to select applicants; 93% (26/28) were worried that video interviews would prevent them from getting a good sense of interviewees and 86% (24/28) were worried that video interviews would prevent them from finding residents who best fit the program (Table 3).

**Table 2:** Applicant survey data before interviews (*n* = 360)

5-Point Likert Questions	Mean ± SD	Median	IQR	Proportion in Agreement
I feel the need to apply to more programs because of video interviews	3.61 ± 1.03	4	3–4	60%
I would rather do in-person interviews	4.48 ± 0.72	5	4–5	91%
I am worried that video interviews will negatively impact my ability to match	3.59 ± 0.98	4	3–4	55%
I am worried that video interviews will not allow me to represent myself as well as in-person interviews would	4.11 ± 0.86	4	4–5	80%
Video interviews provide more flexibility for my schedule	4.34 ± 0.65	4	4–5	93%
I believe that the financial burden of in-person interviews is relieved by video interviews	4.3 ± 0.72	4	4–5	90%
I feel that in-person interviews give a better representation of the culture of a residency program	4.62 ± 0.63	5	4–5	95%
I am more likely to accept a video interview because of reduced cost of travel/other expenses	3.89 ± 1.02	4	3–5	73%
I feel this survey adequately assessed my perceptions of video interviews	3.91 ± 0.62	4	3–4	80%

All 5-point Likert Question answer choices ranged from Strongly Disagree (1), Disagree (2), Neutral (3), Agree (4), Strongly Agree (5). These numerical translations were used to calculate response averages and standard deviation

*IQR* Interquartile Range, *SD* Standard Deviation

**Table 3:** Resident and attending survey data before interviews (*n* = 28)

5-Point Likert Questions	Mean ± SD	Median	IQR	Proportion in Agreement
I would rather conduct in-person interviews	5	5	4–5	100%
I am worried that video interviews will prevent applicants from finding the best program for themselves	4.03 ± 0.99	4	4–5	79%
I am worried that video interviews will prevent programs from finding residents who fit the program best	4.14 ± 0.75	4	4–5	86%
I am worried that the program will not get as good a sense of interviewees because of video interviews	4.21 ± 0.68	4	4–5	94%
I am worried that video interviews will impact how applicants rank our program	3.92 ± 1.02	4	3–5	71%
I am worried that video interviews will not allow applicants to represent themselves as well as in-person interviews	3.96 ± 0.693	4	4–4	82%
I am worried that the absence of away rotations will not allow us to rank the best candidates possible	4.42 ± 0.690	5	4–5	89%
I expect video interviews to answer all of the questions applicants have about our program	2.78 ± 1.07	2	2–4	39%
Video interviews provide more flexibility for my schedule	3.67 ± 0.90	4	3–4	61%
I believe the financial burden of in-person interviews is relieved by video interviews	4.5 ± 0.57	5	4–5	96%
I feel this survey adequately assessed my perceptions of video interviews	3.71 ± 0.76	4	3–4	68%

All 5-point Likert Question answer choices ranged from Strongly Disagree (1), Disagree (2), Neutral (3), Agree (4), Strongly Agree (5). These numerical translations were used to calculate response averages and standard deviation

*IQR* Interquartile Range, *SD* Standard Deviation

When asked to consider potential cost- and time-saving benefits of video interviews, 93% (335/360) of applicants indicated that the format would provide more flexibility for their schedule and 90% (324/360) believed that it would relieve the financial burden of in-person interviews. Additionally, 61% (220/360) of applicants applied to more than 81 programs, 60% (216/360) responded that they felt the need to apply to more programs because of video interviews, and 73% (263/360) responded that they were more likely to accept a video interview because of reduced cost of travel and other expenses. After interviews, students ended up spending less money on video interviews than they thought they would (*p* < 0.001). After interview season, applicants were also more likely to accept a video interview because of reduced cost compared to in-person interviews (80% vs 92%, *p* < 0.01).

Students’ perceptions towards video interviews improved in several categories after interviews (Table 4). Fewer students indicated that in-person interviews were superior to video interviews with regards to representing culture (92% vs 96%, *p* = 0.02) and providing a sense of the residency program (84% vs 100%, *p* = 0.01). Furthermore, student favorability toward a hybrid interview style improved (68% vs 52%, *p* < 0.01). The favorability toward video interviews improved among interviewees but not to a significant degree (4% vs. 16%, *p* = 0.22).

**Table 4:** Comparison of interviewee survey data before and after interviews (*n* = 25)

5-Point Likert Questions	Mean Before (Average ± SD)	Mean After (Average ± SD)	Median, IQR Before	Median, IQR After	*p*-value
I feel the need to apply/I actually applied to more programs because of video interviews	3.64 ± 1.19	2.72 ± 1.18	4, 3–4	3, 2–4	0.0027
I believe that the financial burden of in-person interviews is/was relieved by video interviews	4.64 ± 0.48	4.52 ± 0.64	4, 4–5	5, 4–5	0.023
I feel that in-person interviews give a better representation of the culture of a residency	4.64 ± 0.56	4.56 ± 0.64	5, 4–5	5, 4–5	0.021
I feel that in-person interviews allow you to get a better sense of a residency program as a whole	4.72 ± 0.45	4.28 ± 0.83	5, 4–5	4, 4–5	0.01
I feel more likely to accept a video interview because of reduced cost of travel/other expenses	3.68 ± 1.12	3.72 ± 0.96	4, 3–5	4, 3–5	0.0084
I would prefer a hybrid interview style with both video and in-person components	3.24 ± 1.17	3.72 ± 1.25	4, 2–4	4, 3–5	0.004
I would rather do in-person interviews than video interviews	4.48 ± 0.57	4.08 ± 0.62	5, 4–5	4, 4–4	0.23
Video interviews provide more flexibility for my schedule	4.36 ± 0.74	4.68 ± 0.55	4, 4–5	5, 4–5	0.11
I feel this survey adequately assessed my perceptions of video interviews	3.92 ± 0.68	4.08 ± 0.48	4, 3–4	4, 4–4	0.001

All 5-point Likert Question answer choices ranged from Strongly Disagree (1), Disagree (2), Neutral (3), Agree (4), Strongly Agree (5). These numerical translations were used to calculate response averages and standard deviation

*IQR* Interquartile Range, *SD* Standard Deviation

After interviews, residents and attendings were less worried about the impact of video interviews on how interviewees rank the program (83% vs. 89% *p* < 0.01), and they agreed less with the statement that in-person interviews allow interviewers to build a better rapport with applicants compared to video interviews (94% vs 83% *p* = 0.01). However, they were more worried that video interviews would prevent programs from finding candidates who best fit the program (94% vs. 100% *p* = 0.02). The perception that video interviews provide more flexibility and convenience for attendings and residents improved after interviews (61% vs. 72%, *p* = 0.18). Compared to before and after interviews, more residents and attendings reported they were worried that the absence of away rotations would not allow the program to rank the best applicants possible (94% vs. 100%, *p* = 0.30). Residents and attendings also reported greater comfort hosting video interviews compared to in-person interviews after interview season (16% vs. 83%, *p* = 0.47). Comparisons of resident and attending data is represented in Table 5.

**Table 5:** Comparison of resident and attending survey data before and after interviews (*n* = 18)

5-Point Likert Questions	Mean Before (Average ± SD)	Mean After (Average ± SD)	Median, IQR Before	Median, IQR After	*p*-value
I would rather conduct in-person interviews	4.44 ± 0.61	4.22 ± 1.003	5, 4–5	5, 4–5	0.43
I think that applicants will/did apply to more programs because of video interviews	4.27 ± 1.02	4.72 ± 0.57	5, 4–5	5, 5–5	0.008
I am worried that video interviews will prevent programs from finding residents who fit the program best	4.33 ± 0.59	4.38 ± 0.61	4, 4–5	4, 4–5	0.02
I am worried that video interviews will impact how applicants rank our program	4.22 ± 0.88	4.05 ± 0.87	4, 3–5	4, 4–5	0.006
I feel that in-person interviews allow me to build better rapport with applicants compared to video interviews	4.38 ± 0.61	4.22 ± 0.88	4, 4–5	4, 4–5	0.01

All 5-point Likert Question answer choices ranged from Strongly Disagree (1), Disagree (2), Neutral (3), Agree (4), Strongly Agree (5). These numerical translations were used to calculate response averages and standard deviation

*IQR* Interquartile Range, *SD* Standard Deviation

## Discussion

This investigation determined that the video interview platform for the 2020–2021 orthopaedic surgery residency interview season was initially perceived as inferior to traditional in-person interviews by applicants, residents and attendings. However, following the interview season, perceptions improved overall, and favorable aspects of the video platform have set the precedent for future interview seasons. Nevertheless, in-person interviews were still heavily favored by the vast majority of survey respondents.

Orthopaedic surgery residencies place a high value on interpersonal interaction and personality fit within a program [23]. Therefore, it is not surprising that initial favorability towards video interviews was low in this survey study. Although the video platform provides a viable alternative to in-person interviews in the right situations, [3, 6, 13, 16–19] a number of concerns were elucidated by the survey responses in this study. Most applicants were worried about their ability to adequately represent themselves with a video interview format and felt that they would not be able to gauge the culture of a residency program as well with video interviews compared to in-person interviews, which aligns well with previous literature [6, 20, 21]. Residents and faculty were similarly concerned about their ability to assess fit using video interviews, a sentiment that significantly strengthened after interviews were conducted.

A strong desire to find applicants who fit well in the culture of a program is commonplace across many specialties. In orthopaedics, this desire is exemplified by the extreme weight placed on visiting rotations, which allow residents and faculty members to assess an applicant’s noncognitive skills, such as the ability to develop interpersonal relationships, communicate, and function as a member of the team [24]. This is integral to both the program and candidate to evaluate how compatible of a fit the applicant is with the culture of a program [23]. Compared to students who did zero visiting rotations, Baldwin et al. found that students who participated in just two away rotations were 60 times more likely to match into orthopaedic surgery [24]. This is highlighted in the responses of the present study, which found that 100% of residents and attendings were worried that the absence of visiting rotations would not allow the program to rank the best candidates possible. These findings illustrate the impact of personal interactions in selecting the most compatible candidates for future residency positions.

Prior to the pandemic, multiple programs and specialties had already experimented with the video interview format with varying levels of success. A study that randomized urology residency applicants to receive either video or in-person interviews found that both applicants and faculty favored using video interviews as an adjunct to in-person interviews, despite the video-interview format being perceived as overall less effective than traditional interviews [17]. Likewise, in a study of gastroenterology fellowship applicants participating in both in-person and video interviews on the same day, 87% (14/16) supported video interviews being offered as an option and 81% (13/16) stated that video interviews met or exceeded their expectations [13]. A family medicine residency program utilized video interviews as a screening tool for applicants and found that the majority of interviewers and applicants thought video interviews should be part of the application process; however, neither applicants nor interviewers felt they should be the only means of interviewing [25]. Likewise, this study demonstrated that applicants were more favorable towards a hybrid interview format and felt less strongly that in-person interviews were better for assessing culture and compatibility after the conclusion of the interview season. Additionally, residents and attendings were less worried regarding the impact of video-interviews on how applicants would rank their program and felt less strongly that in-person interviews were superior to video-interviews in building rapport. Although in-person interviews were still heavily preferred by the majority of respondents, these findings suggest that the 2020–2021 residency interview season may have increased acceptability towards the future use of video interviews in the application process.

Several aspects of the video interview format were favorable among participants, which are worthy of mention. Applicants reported that their financial burden of interviewing was significantly reduced compared to historical costs of in-person interviews, with over half of applicants reporting that they spent under $500 and the majority of applicants (80%) spending less than $2000. Compared to previous years, Fogel et al. reported a mean of $7119 among 43 orthopaedic residency applicants [14]. Video interviews also saved the program money and on average, they were perceived as more convenient among all groups. With regards to convenience, residents and faculty experienced fewer interruptions in daily workflow and medical students experienced fewer conflicts with clerkship schedules.

A potentially negative by-product of this convenience and flexibility resulted in applicants applying to and interviewing at a greater number of programs, which is a theme of video interviews that is commonplace in the literature [3, 13, 14, 18]. The ease of electronic application submission was the initial catalyst in dramatic increases of applicants across all specialties, with some specialties nearly doubling their application number [26]. While video interviews potentially enhance the applicant’s reach, it may be at detrimental costs. Weissbart et al. determined that applying to more programs does not improve match rate; rather, the authors suggest that this has increased the selectiveness and the competitiveness of certain specialties [27]. Therefore, it is possible that the already competitive field of orthopaedic surgery may become even more so if video interviews are offered in the future.

There are several limitations to this study. Our investigation took place at a single institution and looked at data over the course of one application cycle. Despite our best effort, response rate of our emailed surveys never broke above 60% so the data presented is not representative of the entire population of applicants to our institution nor the entire population of faculty in the orthopaedic surgery department. Many students who responded to our surveys had never experienced in-person interviews for orthopaedic surgery residency in the past, so they had no baseline to compare their video interviews to. Additionally, those who applied to our institution are not representative of the entire population of orthopaedic surgery residency applicants across the United States which limits generalization of our data.

## Conclusions

In-person interviews for Orthopaedic Surgery Residency are perceived as superior and are preferred among the overwhelming majority of applicants, residents, and interviewers. Nevertheless, perceptions toward video interviews improved in certain domains after interview season, identifying potential for areas of improvement and alternative interview options for future applicants.

## Data Availability

The datasets generated and/or analyzed during the current study are not publicly available due to the policies of the American Association of Medical Colleges and the conditions agreed upon in AAMC approval of this study. As such, the raw datasets generated and/or analyzed during the current study are not available from the corresponding author on request per AAMC guidelines and regulations.

## Abbreviations

AAMC: American Association of Medical Colleges
COVID-19: Coronavirus Disease 2019
IRB: Institutional Review Board
UT: Utah
ERAS: Electronic Residency Application System
IBM: International Business Machines Corporation
NY: New York
SPSS: Statistical Package for the Social Sciences
